# Potential of quantitative X‐ray imaging from photon‐counting CT: A novel analysis based on effective atomic number and physical density

**DOI:** 10.1002/acm2.70673

**Published:** 2026-07-07

**Authors:** Takashi Asahara, Rina Nishigami, Natsumi Kimoto, Mana Mitani, Yusuke Morimitsu, Noriaki Akagi, Fumiyo Higaki, Toshihiro Iguchi, Takao Hiraki, Hiroaki Hayashi

**Affiliations:** ^1^ Department of Radiological Technology, Faculty of Health Sciences Okayama University Okayama Japan; ^2^ Graduate School of Medical Sciences Kanazawa University Kanazawa Ishikawa Japan; ^3^ Department of Radiological Science, Faculty of Health Sciences Junshin Gakuen University Fukuoka Japan; ^4^ Division of Radiological Technology, Medical Support Department Okayama University Hospital Okayama Japan; ^5^ Department of Radiology, Medical Development Field Okayama University Okayama Japan; ^6^ Department of Radiology, Faculty of Medicine, Dentistry and Pharmaceutical Sciences Okayama University Okayama Japan; ^7^ College of Transdisciplinary Sciences for Innovation Kanazawa University Kanazawa Ishikawa Japan

**Keywords:** computed tomography, density image, photon‐counting, virtual monoenergetic image, X‐ray diagnosis

## Abstract

**Background:**

Conventional CT assessment of lung lesions is based mainly on morphological features. CT values represent relative X‐ray attenuation that does not directly reflect tissue composition and/or physical density. Quantitative imaging using photon‐counting CT (PC‐CT) has the potential to provide quantitative parameters, such as effective atomic number (Z_eff_) and effective physical density (ρ_eff_).

**Purpose:**

The purpose is to develop and demonstrate the potential of an algorithm that generates both the Z_eff_ and ρ_eff_ images, derived directly from virtual monoenergetic images (VMIs) acquired with a clinical PC‐CT system.

**Methods:**

The ρ_eff_ image was generated by fitting a database of mass attenuation coefficients (μ/ρ) to the measured linear attenuation coefficients (μ), which were obtained from two VMIs at 70 and 100 keV. As an effective material, Z_eff_ information was also determined and fed back into the ρ_eff_ calculation. An in‐house low‐density phantom was scanned for quantitative evaluation of ρ_eff_. In addition, to demonstrate the applicability of our procedure to actual clinical imaging, representative chest PC‐CT images from patients were analyzed.

**Results:**

In experiments using low‐density phantoms, the CT values did not necessarily correlate with Z_eff_ values, but they showed a very good correlation with the ρ_eff_ values. The ρ_eff_ values calculated using our procedure correlated well with the correct values of ρ. A relative root mean square error of ρ_eff_ calculation was 5.5%. Furthermore, we found that in pulmonary diagnosis, image contrast information from ρ_eff_ makes it easier to identify and distinguish lesions compared to that from Z_eff_.

**Conclusions:**

The proposed procedure directly generated Z_eff_ and ρ_eff_ images from PC‐CT data and enabled quantitative interpretation of CT value in terms of elemental composition and physical density. In this study, the feasibility of generating Z_eff_ and ρ_eff_ images was demonstrated.

**Abbreviations**
μ: linear attenuation coefficient
μ/ρ: mass attenuation coefficient
AEC: automatic exposure control
BMD: bone mineral density
CNR: contrast‐to‐noise ratio
CT: computed tomography
CTDI_vol_
: volume CT dose index
DE‐CT: dual‐energy CT
FOV: field of view
HA: hydroxyapatite
HU: Hounsfield unit
IARC: International Agency for Research on Cancer
NIST: National Institute of Standards and Technology
PC‐CT: photon‐counting CT
QCT: quantitative CT
QIR: quantum iterative reconstruction
Qr: quantitative regular
RMSE: root mean square error
ROI: region of interest
RSS: residual sum of squares
SD: standard deviation
VMI: virtual monoenergetic image
Z: atomic number
Z_eff_
: effective atomic number
ρ: physical density
ρ_eff_
: effective physical density

## 1 INTRODUCTION

According to the GLOBOCAN 2022 statistics published by the International Agency for Research on Cancer (IARC), lung cancer accounted for approximately 18.7% of all cancer‐related deaths worldwide, making it the leading cause of cancer mortality.^1^ Early detection of lesion is important,^2^, ^3^ and therefore chest X‐rays or computed tomography (CT) serve as standard screening and diagnostic tools.^4^, ^5^

Traditional CT diagnosis primarily relies on morphological features such as tumor shape, size, and margin characteristics. However, this qualitative assessment has limitations in estimating the malignancy and nature of tumors because image‐based density differences do not necessarily reflect the tissue properties and/or components. In particular, pulmonary tumors often contain complex mixtures of necrotic, fibrotic, hemorrhagic, or calcified components,^6^ which are difficult to evaluate using qualitative imaging alone.^7^ Therefore, the use of quantitative imaging, which can assess tissue composition and density, may provide additional information for characterizing pulmonary lesions.^7^, ^8^, ^9^, ^10^

Compared to conventional CT, photon‐counting CT (PC‐CT) that is recently developed^11^, ^12^ offers the following advantages; 1. higher spatial resolution at sub‐millimeter levels,^13^, ^14^ 2. lower noise (e.g. approximately 30% reduction),^13^, ^15^ and 3. an improved contrast‐to‐noise ratio (CNR).^11^, ^16^ In addition, it has energy‐resolving capability and therefore the potential to allow simultaneous acquisition of structural and quantitative data by analyzing the attenuation quantity for each photon energy.^17^, ^18^, ^19^, ^20^

Using PC‐CT and dual‐energy CT (DE‐CT), several methods have been proposed to calculate physical quantities such as effective atomic number (Z_eff_),^21^, ^22^, ^23^ electron density relative to water ($\rho_{e}^{W}$),^24^, ^25^, ^26^, ^27^, ^28^ and bone mineral density (BMD).^29^, ^30^ These approaches, summarized in Table 1, fall into two categories: calibration‐based methods using reference materials and direct computational approaches based on theoretical models. In many direct computational approaches, material decomposition is performed by assuming two and/or three basis materials such as water, iodine, calcium, and hydroxyapatite. Therefore, the estimated physical quantities depend on the selected basis‐material combinations and calibration condition, which may limit their applicability to heterogeneous and/or unknown component tissues. Although conventional CT values are relative quantities representing relative X‐ray attenuation and do not provide direct physical meaning, quantitative parameters express tissue composition and density as physical quantities, allowing the objective assessment of pathology. Quantitative imaging may provide additional information for interpreting whether CT value changes are primarily associated with differences in elemental composition or physical density.

**TABLE 1:** Comparison of various quantitative image analyses using PC‐CT or DE‐CT.

Quantity	Notation	Procedure (example)	Reference
Effective atomic number	$Z_{\mathrm{eff}}$	Calibration	21
		$\frac{{\left(\mu /\rho \right)}_{H}}{{\left(\mu /\rho \right)}_{L}}=\frac{\mu_{H}}{\mu_{L}}\to Z_{\mathrm{eff}}$	22, 23
Electron density relative to water	${\rho_{e}}^{w}$	Calibration	24, 25
		$\mu_{k}\approx c_{k}N_{e}+p_{k}\tau$ ^a^	26, 27, 28
Bone mineral density	$\rho_{\mathrm{BM}}$	QCT (Calibration)	29
		$\mu_{k}\approx \rho_{\mathrm{HA}}{\left(\mu /\rho \right)}_{\mathrm{HA},k}+\rho_{\mathrm{soft}}{\left(\mu /\rho \right)}_{soft,k}$ ^b^	30
Effective physical density	$\rho_{\mathrm{eff}}$	$\left(\mu_{L},\mu_{H},Z_{\mathrm{eff}}\right)\to \rho_{\mathrm{eff}}$	This study

^a^ This calculation uses the expression of $\mu_{k}$ at energy *k* in terms of the sensitivity coefficients for Compton scattering $\left(c_{k}\right)$, the photoelectric effect $\left(p_{k}\right)$, and the energy‐dependent coefficient related to the photoelectric effect, τ, to estimate the parameter $N_{e}$ for generating the ${\rho_{e}}^{w}$.

^b^ This method assumes that $\mu_{k}$ is composed of hydroxyapatite (HA) and soft tissue, and the corresponding densities $\rho_{\mathrm{HA}}$ and $\rho_{\mathrm{soft}}$ are estimated based on a bone mineral density image.

The purpose of this study is to propose an algorithm that calculates both Z_eff_ and effective physical density (ρ_eff_) from virtual monoenergetic images (VMIs) obtained with PC‐CT. Our procedure does not involve converting CT values using a specific calibration phantom, but is based on a novel quantitative image calculation approach. Our method is applicable to each voxel and does not require prior region extraction and/or material identification. This paper describes the methodology of a newly developed algorithm. To validate the methodology, experiments were conducted using an in‐house low‐density phantom. Furthermore, representative clinical images were analyzed to demonstrate applicability of the proposed algorithm to clinical PC‐CT data.

## 2 MATERIALS AND METHODS

### 2.1 Algorithm to generate Z_eff_ and ρ_eff_ images

The CT value is defined by the following formula, $CT\;value\;\left[\mathrm{HU}\right]=\frac{\mu -\mu_{w}}{\mu_{w}}\;\times 1000$, where μ and μ_w_ are the linear attenuation coefficients of the object and water (as the reference), respectively. The CT value reflects the μ value of the object of interest. μ value is the attenuation rate of X‐rays per 1 centimeter and can be expressed as μ/ρ×ρ, where μ/ρ is the mass attenuation coefficient that depends on the atomic number (Z) and ρ is the physical density. As the equation indicates, the CT value reflects a combined influence of both Z and ρ.

**FIGURE 1:**
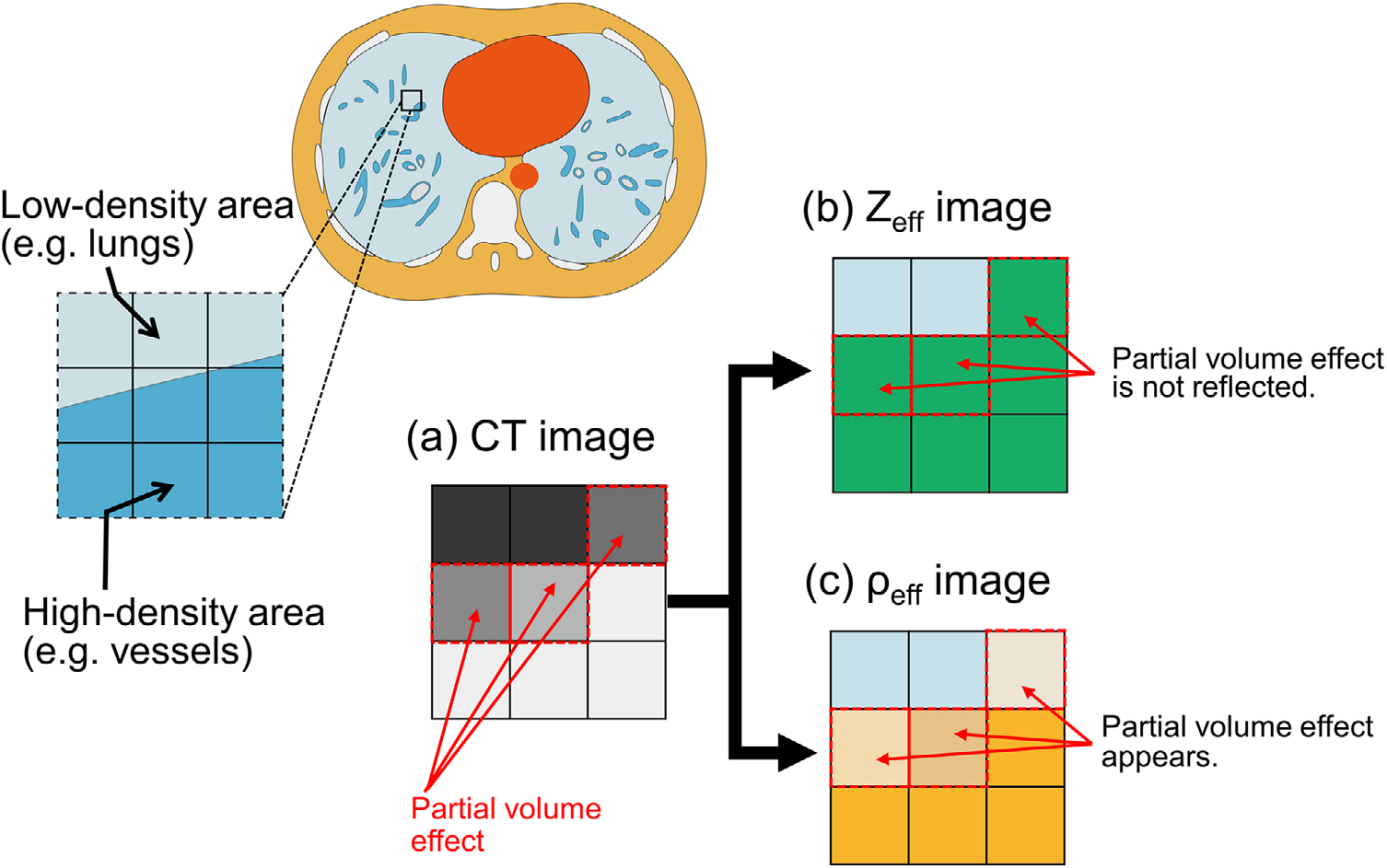
Conceptual diagram of (a) conventional CT, (b) effective atomic number (Z_eff_), and (c) effective physical density (ρ_eff_) images. The figure illustrates how partial volume effects are represented differently in CT, Z_eff_, and ρ_eff_ images.

In this paper, the effective atomic number of a composite material is defined as “Z_eff_”,^9^, ^22^ and the corresponding physical density is defined as “ρ_eff_”. Figure 1 illustrates the conceptual differences among conventional CT, Z_eff_, and ρ_eff_ images. The close‐up view shows a 3 × 3 pixel region containing both low‐density and high‐density areas. In a conventional CT image (Figure 1a), the partial volume effects^31^ influence the pixel values because CT value reflects mixed contributions of elemental composition and physical density within the pixel. In contrast, partial volume effects are represented differently in Z_eff_ and ρ_eff_ images because the proposed procedure separately estimates elemental composition and physical density.

Z_eff_ imaging is a visualization technique of an elemental map, in which composite materials are approximated as a single material with a hypothetical atomic number. This calculation related to element simplification is performed within a given pixel, and Z_eff_ does not contain information about the amount of substances. If heterogeneous substances exist within a pixel because of partial volume effects, the Z_eff_ is calculated from the average amount of elements. Therefore, as shown in Figure 1b, the Z_eff_ in the pixel reflecting the partial volume effect is depicted as the value that strongly reflects the influence of the higher‐density region. In other words, the partial volume effect is less apparent in the Z_eff_ image between the same material.

On the other hand, the ρ_eff_ images in Figure 1c represent physical quantities that simultaneously reflect both the weight and the total amount of the elements contained in the pixel. Accordingly, the partial volume effect is appropriately represented in the ρ_eff_ image, as shown in Figure 1c. In principle, our procedure enables the calculation of ρ_eff_ for individual pixels where Z_eff_ can be determined under the radiation physics theory. However, our procedure cannot be applied in extreme cases where Z_eff_ calculations fail; for example, when the object consists of different atomic number materials with multiple K‐shell and L‐shell absorption edges. In this study, each pixel is represented as an effective material composed of mixed elemental components, and the corresponding effective physical density is estimated.

**FIGURE 2:**
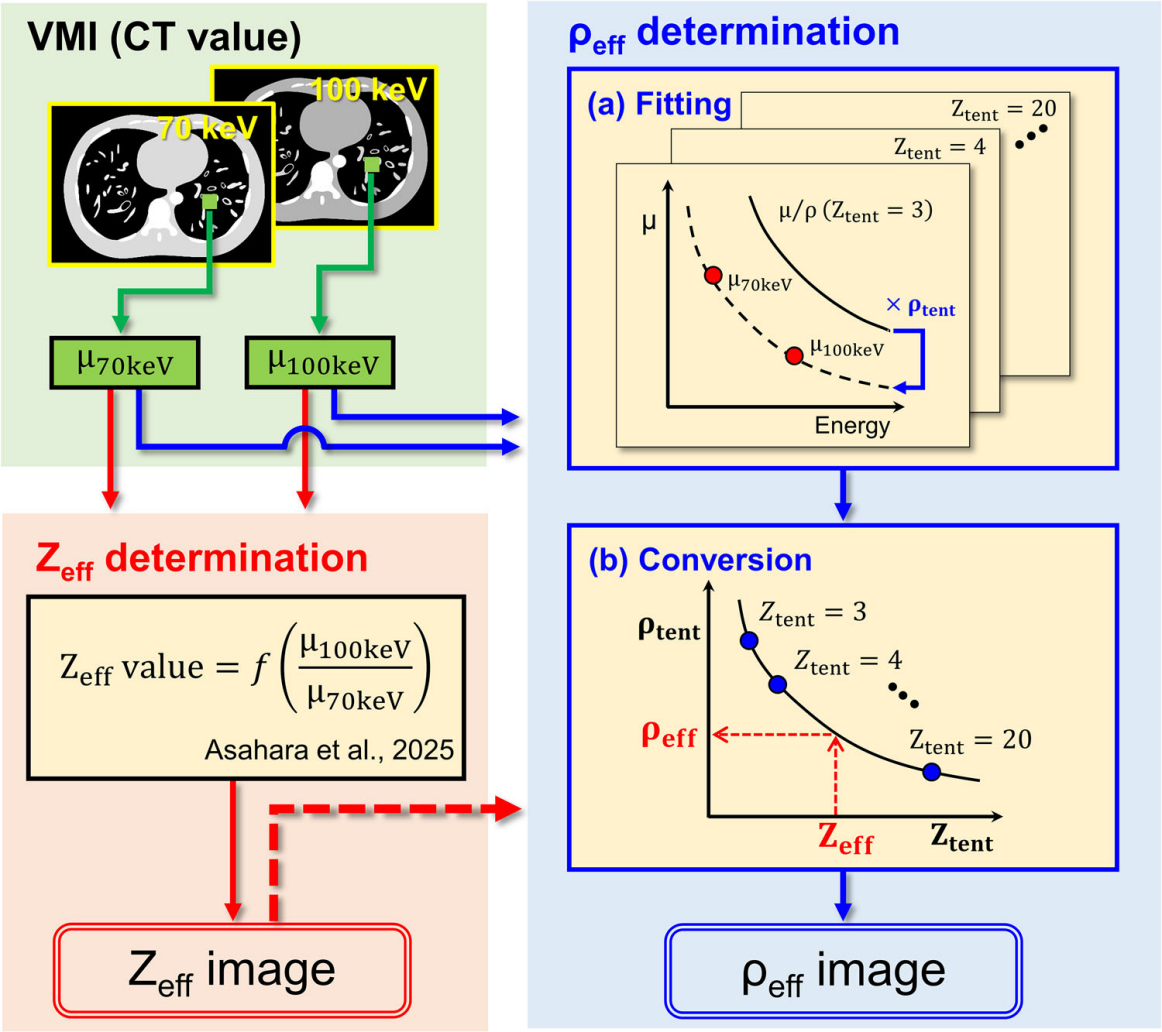
Analysis algorithm for generating effective atomic number (Z_eff_) and effective physical density (ρ_eff_) images from virtual monoenergetic images (VMIs). In the process of ρ_eff_ determination, (a) tentative ρ_eff_ (ρ_tent_) is derived by fitting μ/ρ values corresponding to a tentative atomic number (Z_tent_) to measured μ, and (b) ρ_tent_ is converted to ρ_eff_ using Z_eff_ information. An algorithm for the determination of Z_eff_ images has been proposed previously^22^, ^23^.

The proposed algorithm for generating the ρ_eff_ image is illustrated in Figure 2. Image analysis was performed using an in‐house program written in Mathematica programming software (Wolfram Research, Champaign, IL, USA). This algorithm extends the previously reported procedure for generating Z_eff_ images from VMIs at two energies,^23^ as shown on the left side of the figure. In this process, the imported VMIs at 70 and 100 keV were converted into linear attenuation coefficients (μ). The ratio of these μ values was then used to determine Z_eff_ as follows:

(1) $$Z_{\mathrm{eff}}\;\mathrm{value}\;=f\left(\frac{\mu_{100\mathrm{keV}}}{\mu_{70\mathrm{keV}}}\right),$$

where *f* is a conversion function. Specifically, $\mu_{100\mathrm{keV}}/\mu_{70\mathrm{keV}}={\left(\mu /\rho \right)}_{100\mathrm{keV}}/{\left(\mu /\rho \right)}_{70\mathrm{keV}}$ value for each element was calculated from the tabulated μ/ρ values,^32^ and the continuous conversion function *f* was obtained by spline‐interpolating them. The Z_eff_ value for each pixel was then determined by converting the measured ratio $\mu_{100\mathrm{keV}}/\mu_{70\mathrm{keV}}$ to this function.

The procedure for calculating ρ_eff_ is shown in the blue area on the right. The basic idea is to fit μ/ρ values corresponding to various atomic numbers to the measured μ values. For this purpose, two measured μ values ($\mu_{E,\exp}:E\;=70100\;\mathrm{keV}\;\mathrm{and}\;\;\mathrm{keV}$) were used. By assuming a tentative atomic number Z_tent_, spline‐interpolating continuous functions of the μ/ρ values with Z_tent_ ($\frac{\mu }{\rho }\left(E,Z_{\mathrm{tent}}\right)$) were fitted to the measured μ values, as illustrated in Figure 2a. Namely, the two experimental data ($\mu_{E,\exp}$) were fitted by minimizing the residual sum of squares (RSS), and then the optimal parameter of $\rho_{\mathrm{tent}}\left(Z_{\mathrm{tent}}\right)$ was determined:

(2) $$\mathrm{RSS}\;=\sum_{E\in \left\{70\;\mathrm{keV},\;100\;\mathrm{keV}\right\}}{\left(\mu_{E,\exp}-\frac{\mu }{\rho }\left(E,Z_{\mathrm{tent}}\right)\times \rho_{\mathrm{tent}}\left(Z_{\mathrm{tent}}\right)\right)}^{2}.$$

The obtained fitting coefficient represents a tentative physical density (ρ_tent_) as a function of Z_tent_. The Z_tent_ range was selected to cover the Z_eff_ values of typical biological tissues (e.g. soft‐tissue: Z_eff_ ≈ 7, bone: Z_eff_ ≈ 13). Therefore, as shown in Figure 2b, ρ_tent_ was interpolated for Z_tent_, and ρ_eff_ was calculated by substituting Z_eff_ into this function.

(3) $$\;\rho_{\mathrm{eff}}=\rho_{\mathrm{tent}}\;\left(Z_{\mathrm{eff}}\right).$$

Our algorithm enables the generation of a ρ_eff_ image by using the Z_eff_ value as a parameter that was determined in advance by equation (1). In this study, each voxel was treated as an effective material composed of mixed elemental components, and Z_eff_ and ρ_eff_ were interpreted as effective quantities within the voxel.

### 2.2 Phantom experiment

**FIGURE 3:**
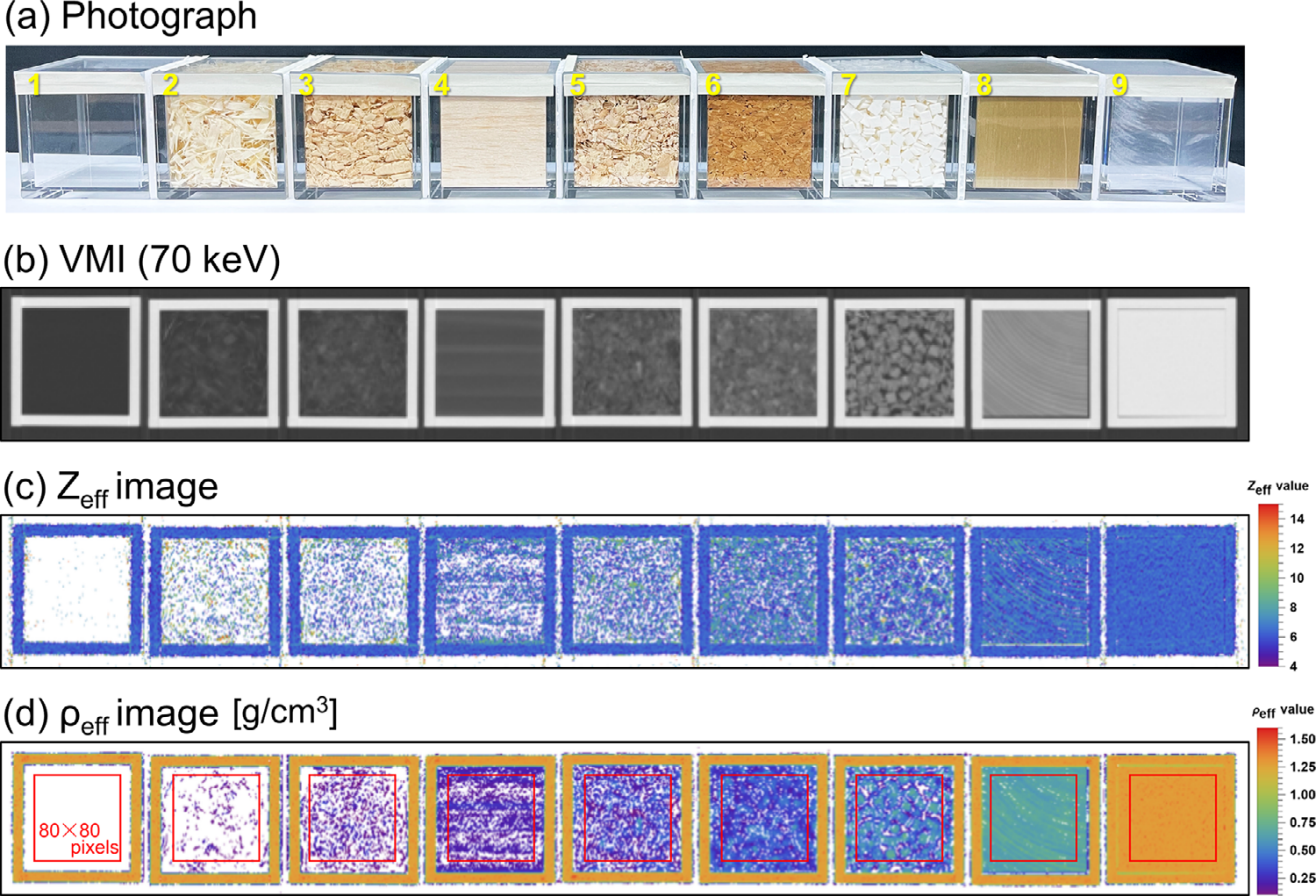
(a) Photograph of the in‐house low‐density phantom used in the experiment to validate the algorithm. (b)–(d) Coronal images of a low‐density phantom obtained with clinical PC‐CT. Pixels in which either μ_70keV_ or μ_100keV_ was less than or equal to 0 were excluded from the analysis. The Z_eff_ image showed similar values across most samples. On the other hand, each ρ_eff_ image is different, which reflects the differences in physical properties between the samples.

We performed a phantom experiment to validate the algorithm for generating ρ_eff_ images. Figure 3a shows the in‐house low‐density phantom. Nine acrylic cubes (50 × 50 × 50 mm^3^) were filled with various low‐density materials. These samples are summarized in Table 2, and the numbers (No.) correspond to the labels in Figure 3a. No. 1 contains only dry air, and the standard air density^33^ was applied as the reference ρ ($\rho_{\mathrm{eff}}^{\mathrm{ref}}$) value. The $\rho_{\mathrm{eff}}^{\mathrm{ref}}$ values for samples No. 2–8 were determined from actual volume and weight. All measurements were performed under conditions controlled at 22 ± 1°C using an air conditioner. For No. 9, acrylic, a well‐known empirical value^34^ was used as the $\rho_{\mathrm{eff}}^{\mathrm{ref}}$ value.

**TABLE 2:** Summary of the samples contained in the phantom and their ρ_eff_ values.

No.	Sample	Reference density: $\rho_{\mathrm{eff}}^{\mathrm{ref}}$ [g/cm^3^]
1	Air	$1.184\times 10^{-3}$ ^a^
2	Aspen wood	0.074^b^
3	Fir wood	0.123^b^
4	Balsa	0.161^b^
5	Birch	0.222^b^
6	Cork	0.296^b^
7	Paperboard chips	0.344^b^
8	Wood	0.580^b^
9	Acrylic	1.19^c^

^a^ This value is the density of dry air at 25°C and 1 atm.^33^

^b^ These were calculated from the actual mass and volume.

^c^ This is a well‐known empirical value.^34^

The low‐density phantom was scanned with a clinical PC‐CT scanner (NAEOTOM Alpha, Siemens Healthineers, Forchheim, Germany). The routine chest CT scan conditions were applied: standard collimation mode (144 rows × 0.40 mm detector) at 140 kV, pitch factor of 0.80, and rotation time of 0.25 s. The exposure setting was an effective mAs of 15, which was determined by the automatic exposure control (AEC) with an image quality level of 80. The volume CT dose index (CTDI_vol_) was 1.72 mGy. VMIs at 70 and 100 keV were reconstructed using the vendor's application. A coronal image plane with a field of view (FOV) of 600 mm × 220 mm with a matrix size of 1536 × 512 was created. A slice thickness was 3.0 mm. A reconstruction kernel of quantitative regular with sharpness level 44 (Qr44), which is routinely used in clinical examinations, was applied. A quantum iterative reconstruction (QIR) strength of 1 was selected. Then, Z_eff_ and ρ_eff_ images were derived using the algorithm described above.

### 2.3 Demonstration using patient images

This study was conducted as a preliminary feasibility investigation to demonstrate that Z_eff_ and ρ_eff_ images can be generated from clinical PC‐CT data. Our research was approved by the ethics review board of the relevant institution. The use of clinical image data from 10 patients was approved, in which 9 males and 1 female ranging in age from 70 to 86 years (mean: 78.2 ± 4.7 y) were included. CT scans were acquired using a clinical PC‐CT system (NAEOTOM Alpha, Siemens Healthineers) under the same routine chest imaging conditions used in the phantom experiment. The exposure setting for each patient was adjusted according to body size using AEC. The average effective mAs was 72.3 ± 22.0, and the corresponding CTDI_vol_ was 12.2 ± 10 mGy. VMIs in the axial plane were acquired at 70 and 100 keV. The FOV was adjusted to the patient's body size and had a diameter of 340–380 mm. The matrix size was 512 × 512. The slice thickness was 5.0 mm.

From the clinical CT datasets of VMIs, the Z_eff_ and ρ_eff_ images were generated using the proposed algorithm. Two representative cases were selected to demonstrate the availability of quantitative image generation. One patient underwent two chest CT scans for pneumonia monitoring during the period for which the use of clinical data was approved by ethical review. The other patient was a typical case with pulmonary nodules. The ROI placement involved defining a square region of interest (ROI) in the lesion and the surrounding normal lung tissue. This was determined manually by a radiological technologist with 8 years of experience. The mean and standard deviation (SD) of CT value, Z_eff_ value, and ρ_eff_ value were measured. Inter‐observer variability was not evaluated because the present study primarily aimed to demonstrate the technical feasibility of the proposed quantitative imaging algorithm.

## 3 RESULTS

### 3.1 Phantom‐based evaluation for ρ_eff_ estimation

Figure 3b–d shows the coronal section plane of the low‐density phantom obtained using PC‐CT. Panels (b), (c), and (d) are the VMI at 70 keV, the Z_eff_ image, and the ρ_eff_ image, respectively. The VMI at 70 keV shows a similar contrast to the conventional CT image,^22^ and the CT values differed for each sample. In the Z_eff_ image, most samples had similar Z_eff_ values. On the other hand, large differences (contrasts) which reflect the physical properties of the investigational samples were observed in the ρ_eff_ images. The red squares indicate ROIs of 80 × 80 pixels to measure the mean and SD values.

**FIGURE 4:**
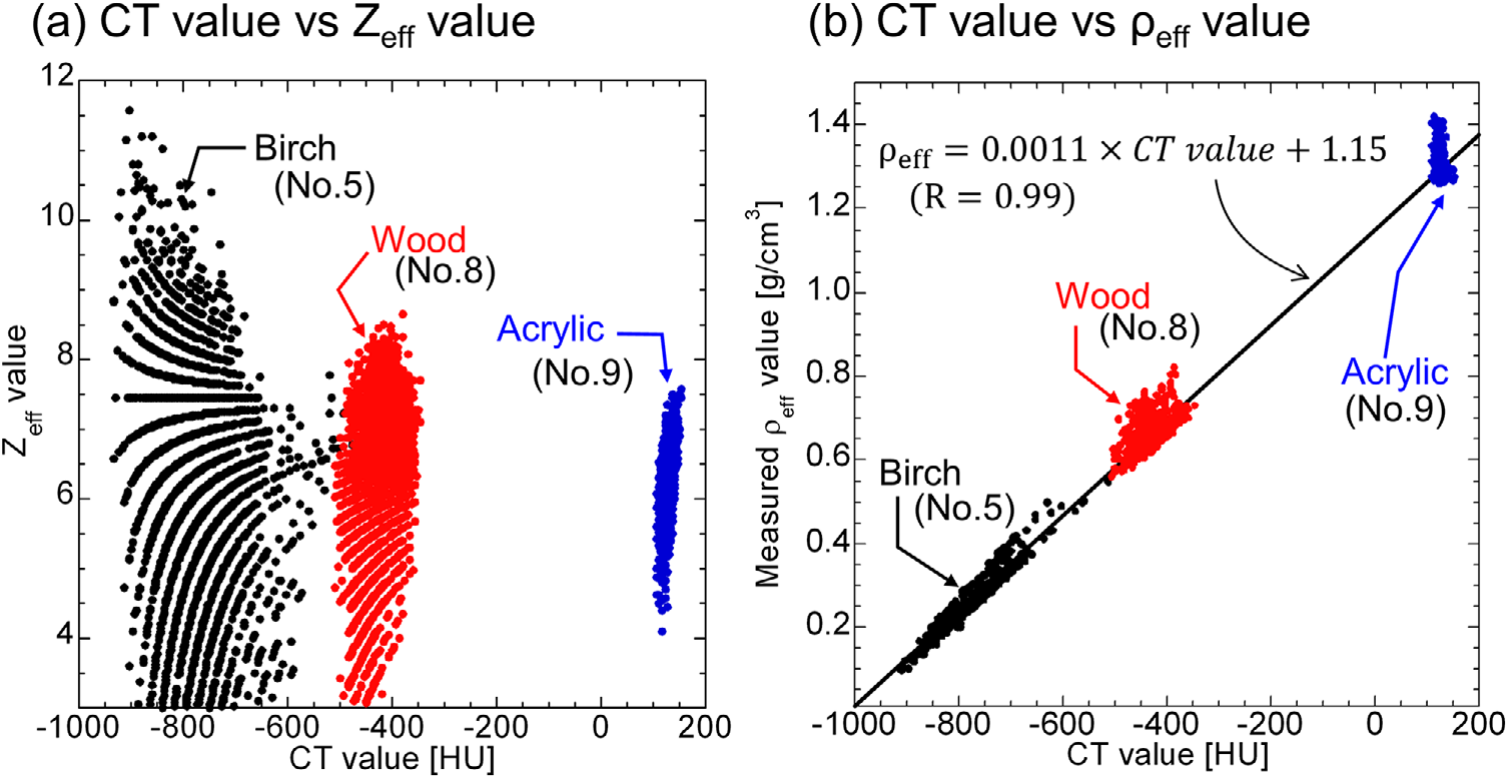
Scatter plots between CT value and quantitative parameters (Z_eff_ and ρ_eff_). (a) CT value versus Z_eff_ value and (b) CT value vs ρ_eff_ value for typical low‐density materials of Birch (No. 5), wood (No. 8), and acrylic (No. 9). Pixel values within 80 ×80‐pixel ROIs were plotted for birch, wood, and acrylic samples. No clear correlation was observed between CT values and Z_eff_, but a strong correlation was observed between CT values and ρ_eff_.

Figure 4 shows pixel‐by‐pixel scatter plots for three representative low‐density materials: birch (No. 5 in Figure 3), wood (No. 8), and acrylic (No. 9). Pixel values of 80 × 80‐pixels in ROIs were plotted. As shown in Figure 4a, the Z_eff_ values do not show a clear correlation with CT values. In contrast, as shown in Figure 4b, the ρ_eff_ values show a strong linear correlation with CT values (*R* = 0.99). The relationship was as follows: $\rho_{\mathrm{eff}}\;\left[g/cm^{3}\right]=0.0011\times CT\;value+1.15$. Because the investigated low‐density materials have similar Z_eff_ values, the CT value strongly reflects the difference in density. However, this relationship is only applicable to samples with similar Z_eff_ values and is not universal. It is known that the various organs of the human body have different Z_eff_ values, and this fact is reflected in producing reference materials such as multi‐energy CT phantoms.^22^, ^23^ However, in the diagnosis of lung diseases, it has been reported that lesions are often mutated cells within the lung tissue.^35^ Based on this fact, it can be assumed that ρ_eff_ images, which have contrast for quantitative changes in substances, are more suitable for diagnosing lung lesions than Z_eff_ images, which have contrast for substance type. To support this hypothesis, an example of clinical data analysis is shown next.

**FIGURE 5:**
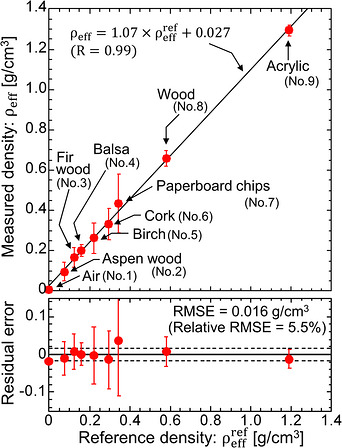
Quantitative evaluation of ρ_eff_ values using the low‐density phantom. Linear regression analysis between reference and measured ρ_eff_ values was performed. The lower graph shows the residual errors from the correlation line. The RMSE was 0.016 g/cm^3^.

Figure 5 compares the $\rho_{\mathrm{eff}}^{\mathrm{ref}}$ value and the measured ρ_eff_ value. Linear regression analysis yielded the following relationship: $\rho_{\mathrm{eff}}=1.07\times \rho_{\mathrm{eff}}^{\mathrm{ref}}+0.027$ with a strong correlation coefficient (*R* = 0.99). We considered that this correlation line is due to methodological uncertainty and systematic errors inherent to the device, such as the accuracy of μ value determination. Therefore, a correction using this relationship was applied to patient data measured with the same CT device. The lower graph shows the residual errors from the correlation line. The root mean square error (RMSE) was 0.016 g/cm^3^, which corresponds to a relative RMSE of 5.5%. This value represents the systematic uncertainty of our analysis procedure. The uncertainty of our procedure depends on the quality of the input data. Specifically, the VMI reconstruction procedure and reconstruction parameters influence the results.

### 3.2 Demonstration using representative clinical images

**FIGURE 6:**
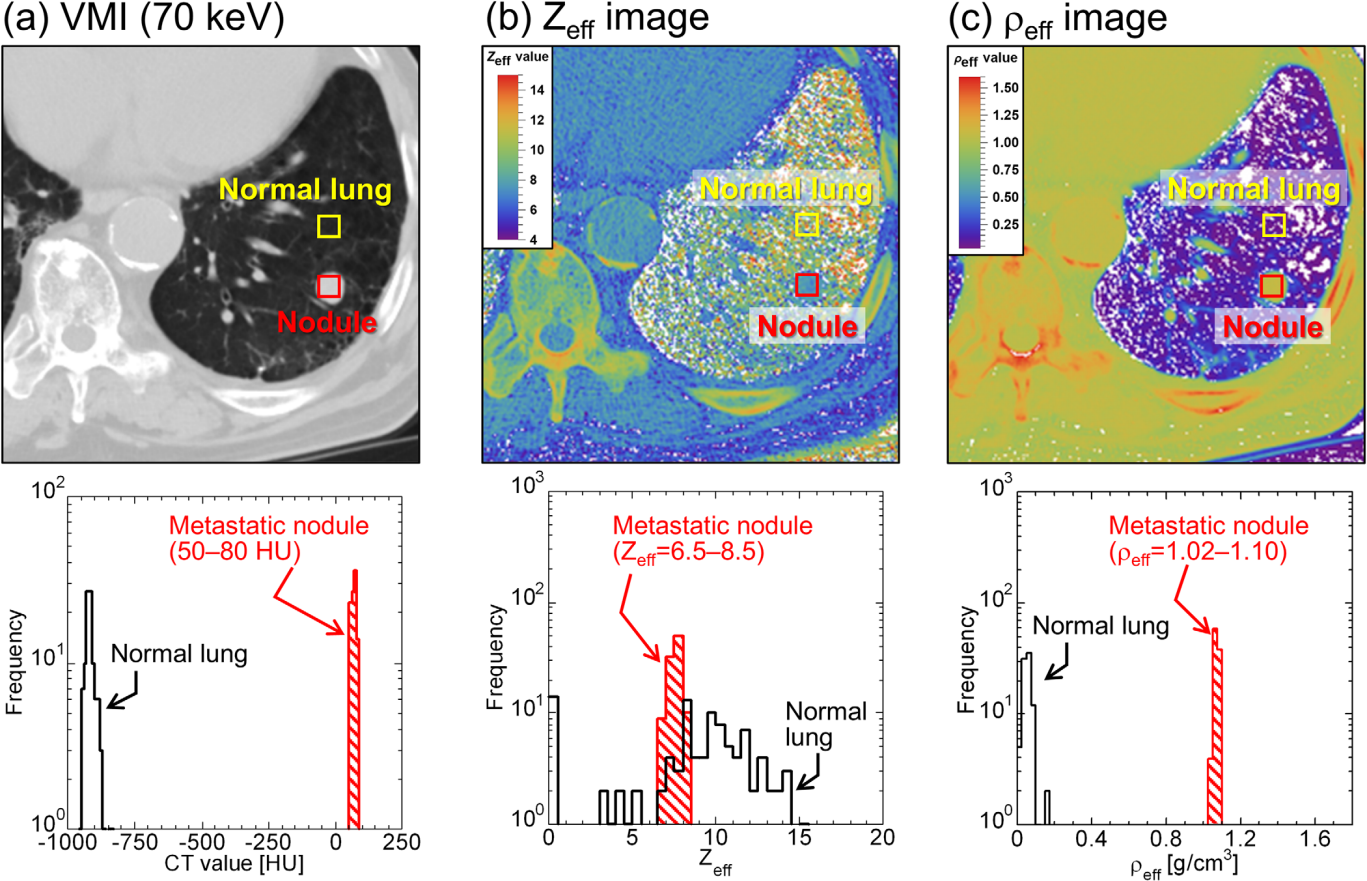
CT image of a patient with lung metastasis. The upper row shows the images, and the lower row shows the quantitative analysis results of the histogram of ROIs within the images. The metastatic nodule showed a similar Z_eff_ value to the surrounding lung tissue, but the ρ_eff_ value was clearly higher.

Figure 6 demonstrates a representative case of lung metastasis in an 85‐year‐old man with a metastatic nodule in the lower left lung. Images (a), (b), and (c) show the VMI, Z_eff_ image, and ρ_eff_ image of the left lung, respectively. The lower graphs are histograms of the CT, Z_eff_, and ρ_eff_ values using the square ROIs at the positions indicated in the images. In the Z_eff_ image, the metastatic nodule showed Z_eff_ values of 6.5–8.5, similar to those of the surrounding normal lung tissue. In contrast, the ρ_eff_ image showed clearly higher values in the nodule (mean ρ_eff_ = 1.07 ± 0.01 g/cm^3^) than those in the surrounding lung tissue (ρ_eff_ = 0.06 ± 0.02 g/cm^3^). A statistically significant difference was also confirmed based on the Wilcoxon rank‐sum test (*p* < 0.0001).

In general, the density of reported normal “lung tissue” is defined as the that of the substance itself, in which air component is excluded; for example, it is reported as 1.05 g/cm^3^ by National Institute of Standards and Technology (NIST).^32^ It should be noted that the measured ρ_eff_ value is the effective density including air and interstitium, and therefore is much smaller than the reported value of the normal lung tissue.

**FIGURE 7:**
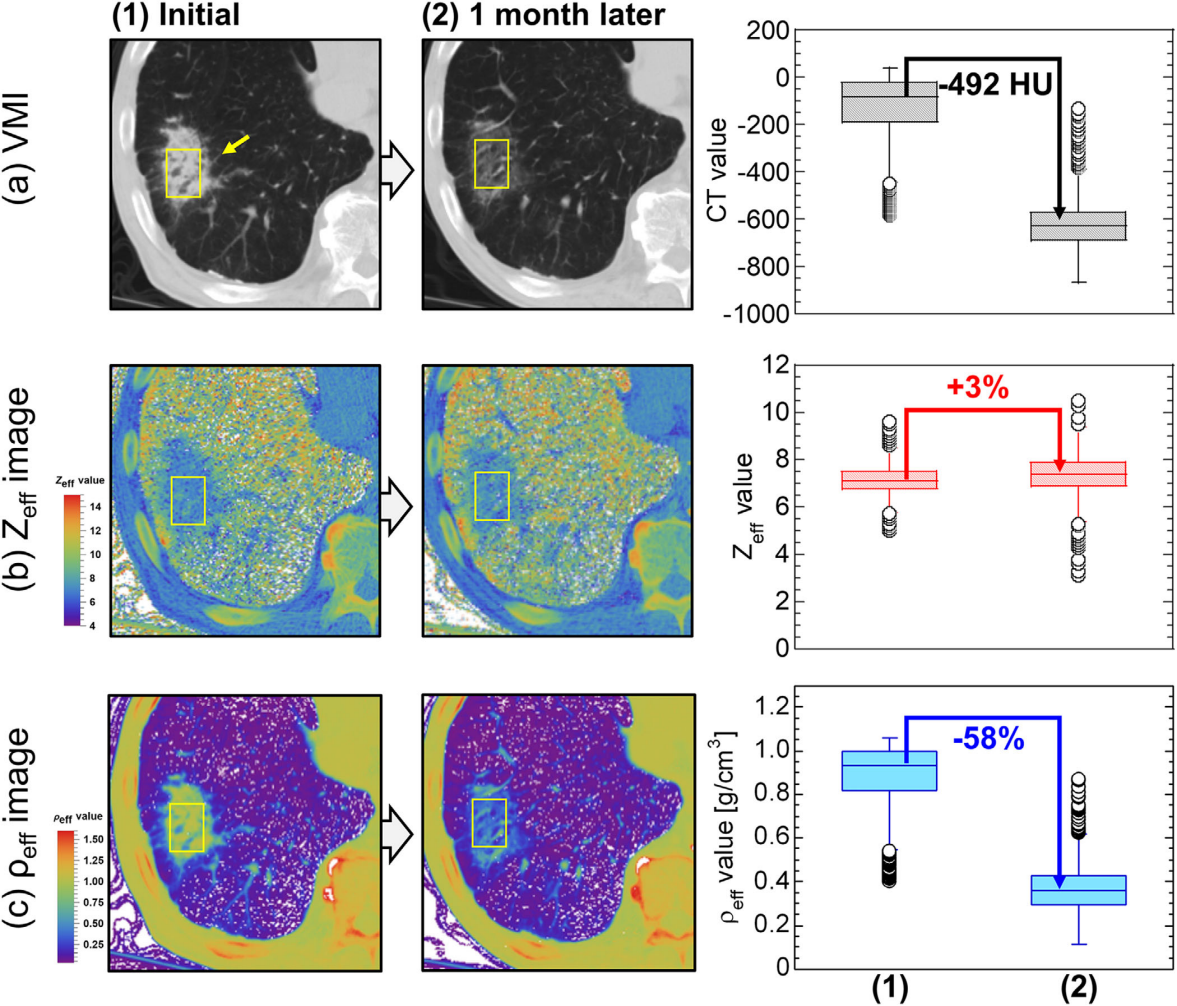
CT images of a patient with pneumonia. (1) The initial and (2) one‐month follow‐up images are shown for (a) VMI, (b) Z_eff_, and (c) ρ_eff_ images. Z_eff_ values showed little change, whereas ρ_eff_ values markedly decreased during lesion regression. The box plots on the right show the quantitative results measured using the ROIs.

Figure 7 shows a representative case of pneumonia in a 78‐year‐old man with the lesion located in the right lung. Panels (a), (b), and (c) show the VMI, Z_eff_, and ρ_eff_ images, respectively. (1) The initial scan and (2) the follow‐up image taken one month later are compared. The right graphs are box plots of the pixel values for the lesion area measured using the ROIs. The inflammatory consolidation area was observed in the initial VMI as a region with relatively large CT values. Follow‐up VMI showed a decrease of −492 HU with regression of the pneumonia. Z_eff_ values on the initial and follow‐up images showed similar values. On the other hand, we observed a marked decrease of −58% in the ρ_eff_ value. This difference was statistically significant based on the Wilcoxon rank‐sum test (*p* < 0.0001).

## 4 DISCUSSION

### 4.1 Impact of quantitative analysis based on Z_eff_ and ρ_eff_

Our algorithm directly generates Z_eff_ and ρ_eff_ images from VMIs obtained with PC‐CT without the need for calibration scanning. The proposed procedure directly derives Z_eff_ and ρ_eff_ images and enables quantitative interpretation of CT values using clinical PC‐CT data. VMIs are expressed as CT values, which are converted from the linear attenuation coefficient of water; however, they essentially represent a physical quantity related to the linear attenuation coefficient (μ) of a substance. As is well known, μ is expressed as the product of the mass attenuation coefficient μ/ρ and density ρ.

(4) $$\mu =\left(\frac{\mu }{\rho }\right)\;\times \rho$$

μ/ρ is a function that changes with atomic number, and this can be expressed as Z_eff_. Namely, it is suggested that VMI expressed in terms of μ can be decomposed into Z_eff_ and ρ_eff_.

In this study, we successfully separated and independently imaged Z_eff_ and ρ_eff_ by solving physics equations without using phantom calibration. To demonstrate that material characterization using ρ_eff_ values can be effective in some cases, we fabricated low‐density phantoms with similar Z_eff_ values and conducted experiments. As shown in Figure 3, the low‐density phantoms showed differences (contrast) in the ρ_eff_ image, which provides information that enables the differentiation among the material. The accuracy of our procedure was also confirmed by these phantoms, and as shown in Figure 5, the relative RMSE of ρ_eff_ values was 5.5%, which represents systematic uncertainty.

A demonstration of lung lesion diagnosis was conducted to show the potential for clinical use of ρ_eff_ imaging. This was because many lung lesions are caused by quantitative changes in existing cells, therefore ρ_eff_ imaging was considered to be suitable for diagnosis. Figures 6 and 7 support this hypothesis, which show significant changes in ρ_eff_ for lesions. However, these are merely demonstrations using small amounts of patient data and do not represent clinical evidence. A larger cohort study is desirable.

### 4.2 Limitations

This study has several limitations. First, the analyzed images contained relatively large noise, indicating the need for optimized image reconstruction and noise‐reduction processing. Although VMI reconstruction includes noise‐reduction processing, it remains unclear whether the applied reconstruction parameters were optimal for the proposed algorithm. We applied the quantitative regular kernel 44 and QIR strength 1 to the analysis. The accuracy of the CT value (i.e., μ) may depend on the kernel type and QIR strength, and it is considered that changes of these parameters to reduce the noise will also affect the accuracy of the analyzed ρ_eff_ value. In this study, all clinical data were acquired using routine protocols with AEC, and our procedure demonstrated feasible performance under these imaging conditions. Recently, the usefulness of low‐dose examinations using PC‐CT has been reported,^13^ but the applicability of our procedure to low‐dose PC‐CT imaging was not evaluated within the scope of the present study. Future implementation may benefit from vendor‐developed applications that directly analyze raw PC‐CT data using optimized reconstruction conditions.

Second, our procedure assumes that each voxel can be approximated as an effective material composed of mixed elemental components. Therefore, heterogeneous microstructures within a voxel that do not produce detectable differences in X‐ray attenuation are not explicitly modeled in the present study. In addition, the VMI combination of 70 and 100 keV was adopted based on the previous research on the use of Z_eff_ images in dental forensic medicine.^23^ Optimization of the combination of energy‐resolved images is also a research topic,^19^, ^36^ and there is a study using a combination of 40 and 190 keV for Z_eff_ images.^22^ The estimated quantitative values may depend on the selected VMI energy pair. There has been little research on the calculation of *ρ*_eff_ value, whith only a few pilot studies.^37^ Further investigation will be necessary to research the optimization of the entire system, including ρ_eff_ images in addition to Z_eff_ images.

Finally, further validation using larger clinical datasets is needed. The objective of the present study was to propose a novel quantitative imaging algorithm and demonstrate its feasibility using phantom experiment and representative clinical images. It should be noted that all examinations were conducted using a single PC‐CT model equipped with a single vendor, and we did not provide general scientific evidence for general PC‐CTs. In addition, the estimated ρ_eff_ values may vary depending on reconstruction conditions such as reconstruction kernel, QIR strength, and low‐dose imaging protocols. Therefore, potential variability across different scanners, reconstruction settings, and institutions has not been evaluated. Further multicenter studies will be necessary to investigate the robustness and generalizability of the proposed procedure.

## 5 CONCLUSION

We proposed a novel procedure for deriving both the effective atomic number (Z_eff_) and the effective physical density (ρ_eff_) images, which can be determined directly from virtual monoenergetic images (VMIs) acquired with a clinical photon‐counting CT (PC‐CT).

We conducted a verification experiment using a low‐density phantom and confirmed that the ρ_eff_ values analyzed by the proposed procedure correlated well with the actual ρ values. A relative root mean square error was 5.5%. Furthermore, a demonstration was performed using clinical data from two patients.

Our procedure enables quantitative interpretation of CT contrast in terms of elemental composition and physical density alongside conventional structural CT evaluation.

## AUTHOR CONTRIBUTIONS

**Takashi Asahara**: Validation; formal analysis; writing – original draft; investigation; data curation; funding acquisition. **Rina Nishigam**: Methodology; software; writing – review and editing. **Natsumi Kimoto**: Methodology; software. **Mana Mitani**: Data curation; investigation. **Yusuke Morimitsu**: Resource; data curation. **Noriaki Akagi**: Resource. **Fumiyo Higaki**: Resource. **Toshihiro Iguchi**: Supervision; writing – review and editing. **Takao Hiraki**: Resource. **Hiroaki Hayashi**: Conceptualization; supervision, project administration; writing – review and editing.

## CONFLICT OF INTEREST STATEMENT

The authors declare no conflicts of interest.

## ETHICS STATEMENT

This study was approved by the institutional ethics committee of Okayama University (Approved No. 2506‐002). The requirement for informed consent was waived due to the retrospective design and use of existing medical data. An opt‐out consent approach was applied for the retrospective data analysis. Written informed consent was obtained from all patients prior to CT examinations.

## Data Availability

The data that support the findings of this study are available from the corresponding author upon reasonable request. The data are not publicly available due to privacy and ethical restrictions.
